# Climate awareness and lower-meat eating among children and adolescents: potential behavioral pathways and contextual conditions

**DOI:** 10.3389/fnut.2026.1967214

**Published:** 2026-09-28

**Authors:** Lu Xing, Xueqi Wu

**Affiliations:** 1 School of Preschool Education, Changsha Normal University, Changsha, China; 2 School of Education, Faculty of Social Sciences and Leisure Management, Taylor's University, Subang Jaya, Malaysia; 3 Taylor's Culinary Institute, Faculty of Social Sciences and Leisure Management, Taylor's University, Subang Jaya, Malaysia

**Keywords:** adolescents, alternative proteins, children, climate awareness, lower-meat eating, school meals, sustainable diets

## Abstract

Climate awareness is increasingly proposed as a motive for lower-meat eating, while research involving children and adolescents spans distinct outcomes, from knowledge and willingness to food provision and measured intake. This Mini Review synthesizes these outcome levels and the conditions that connect them. A targeted structured search of Web of Science and Scopus identified surveys, qualitative studies, message and food-education interventions, family and school provision studies, and nutritional analyses. Evidence was organized as climate-linked motivational, proximal behavioral, and intake-association evidence; provision- and intake-change evidence; and contextual or feasibility evidence. Climate-related motives coexist with health, taste, identity, convenience, and social norms. One preregistered study comprising two randomized message experiments reported changes in immediate sustainable choices and intentions. A cross-sectional youth analysis linked climate attitudes with current intake, retrospectively reported reduction, and future intention, whereas a provision-based family trial supplied the clearest within-study change in meat intake. Family gatekeeping, school-food systems, product acceptability, culture, affordability, nutrition, and equity shape opportunities across these routes. Age changes opportunities for dietary agency: decision control may be adult-led, shared, or youth-led within age groups and settings. Climate awareness therefore occupies one motivational position in a multilevel evidence map. Connecting motivation with acceptable, accessible, and nutritionally adequate food environments provides a testable framework for sustained dietary change.

## 1 Introduction

Sustainability-oriented dietary guidance increasingly calls for limits on some meat consumption (1). At the same time, meat contributes materially to children's and adolescents' protein and several micronutrient intakes in European dietary data (1). The relevant public-health question is how climate-related motivation enters decisions made within families, schools, markets, and institutions while preserving dietary quality.

Children encounter this question within food systems largely organized by adults. Caregivers shape purchasing, preparation, portions, and repeated exposure; schools control much of the weekday food environment; and available products define what can be selected and eaten. Young people's participation in these decisions often expands with age, while actual control varies within every age group. Younger children may influence household purchases, and adolescents may remain dependent on family budgets or fixed school menus. Development changes opportunities for agency, negotiation, and social influence across these settings.

The literature uses several outcomes that occupy different positions in a behavioral pathway. In this review, climate awareness refers to knowledge, recognition, or concern about the environmental implications of food. Attitudes, intentions, and willingness describe motivational readiness. Acceptance and single choice describe product liking, willingness to try, or one immediate selection. Provision and purchasing describe what households or institutions make available. Current or habitual intake describes dietary status at one assessment. Meat-intake change requires a baseline, comparator, or explicit within-study change measure. Persistence requires follow-up beyond the active intervention and is more informatively assessed when compensatory intake outside the observed setting is also measured. Alternative-protein acceptance marks a substitution opportunity; evidence of meat displacement requires a corresponding measure of consumption. “Sustainable protein choice” is used as the broad category encompassing these distinct outcomes.

Two recent syntheses provide foundations for this distinction. One reviews adolescents' perceptions of sustainable diets and school-based interventions (2), while another maps parents' and children's perceptions of traditional and novel plant-based meat alternatives (3). The present Mini Review extends this landscape by aligning studies with the highest outcome they observe and by examining the conditions surrounding movement from motivation to repeated intake.

The review asks: at which behavioral stages is climate awareness associated with meat-related dietary outcomes among children and adolescents, and how do developmental and contextual conditions shape links with current intake, within-study change, and persistence? Its central proposition is that climate awareness occupies a motivational position within a multilevel evidence map. Food-decision control, family practice, school provision, social norms, product acceptability, material access, and the nutritional quality of available replacements shape the available routes to food choice and intake. This outcome-based synthesis contributes a context-sensitive account of dietary agency and a set of intervention hypotheses that can be evaluated with meat-specific and repeated behavioral measures.

## 2 Search and synthesis approach

We conducted a targeted structured search of Web of Science Core Collection and Scopus on 12 August 2026 for English-language journal Articles and Reviews published from 2000 through the search date. The original query combined child and adolescent terms with meat consumption or reduction, vegetarian, vegan, or flexitarian diets, meat alternatives, and alternative proteins, together with climate, sustainability, environmental-awareness, carbon-footprint, greenhouse-gas, or planetary-health concepts. A sensitivity search added youth, pediatric/paediatric, infant/toddler, parent/caregiver/family, childcare and school-meal settings, plus plant-based diet/protein and sustainable-protein terms. Complete strings, database fields, proximity operators, and eligibility rules are reported in Supplementary Tables S1–S3.

The original search returned 79 Web of Science and 81 Scopus records; the sensitivity searches returned 140 and 141 records before deduplication. Across both searches, 441 raw records formed 220 unique records after DOI and normalized-title matching, of which 199 met the year, language, and document-type limits. Evidence-role screening retained an auditable pool of 57 records: 22 child- or adolescent-relevant core records, 30 nutrition, environmental-feasibility, affordability, procurement, or implementation records, two secondary reviews used for landscape orientation, and three background records. Thirty-five records were selected for manuscript claims and underwent full-text verification; the remaining 22 are retained with their screening role and a record-specific citation-selection reason. Selection for citation required either a child/adolescent-specific finding or directly relevant family- or school-setting evidence, together with a non-redundant contribution to a defined outcome stage or contextual condition and full-text support for the manuscript claim. Screening-stage exclusion counts and reasons are reported in Supplementary Tables S2–S2b.

The 35 cited records were coded along two descriptive dimensions. The first recorded study design and procedures, including randomization, comparison conditions, sampling, measurement, follow-up, and setting. The second recorded outcome directness along seven stages: Stage 1, awareness and attitudes; Stage 2, intentions and willingness; Stage 3, acceptance or a single choice; Stage 4, provision or purchasing; Stage 5, current or habitual meat intake; Stage 6, change from a baseline, comparator, or explicit within-study measure; and Stage 7, persistence at follow-up. Separate fields record whether change was meat-specific, the number of measurement occasions, follow-up duration, and whether a child- or adolescent-specific outcome was available. Five evidence-role codes distinguish climate-linked motivational evidence, climate-linked proximal behavioral evidence, climate-linked intake associations, provision- or intake-change evidence, and contextual or feasibility evidence. The remaining 22 records retain screening-scope and citation-selection fields without claim-level coding. The appraisal reports design features and supported inference without assigning a formal risk-of-bias score or summary quality grade.

One reviewer completed title-and-abstract screening and initial evidence-role coding. All 35 cited records subsequently underwent full-text verification using page- or result-section evidence cards that linked each manuscript claim to its source. Supplementary Tables S4–S6 report the design features, outcome stage, evidence role, claim-level support decision, and full-text locator for every cited record. DOI-level overlap between the two landscape reviews and eligible primary studies was audited so that primary studies carried the main inferential weight. The targeted design supports a structured evidence map across heterogeneous designs, exposures, outcomes, and follow-up periods.

## 3 Evidence across outcome stages and contexts

### 3.1 Climate-linked motivational evidence

The most visible climate-linked evidence concerns awareness, motives, attitudes, and intentions. In a survey of 487 young people aged 17–18 years, acceptance of climate science was high, whereas uptake of several high-impact actions was lower; descriptive norms predicted intentions across the behaviors assessed, including dietary action (4). Its meat-related endpoint lies at the level of intention and current-action reporting. Among New Zealand adolescents, “nice, normal, necessary, and natural” rationalizations varied by meat-consumption pattern and sex, while environmental motives were present alongside health, habit, and social meanings (5). These findings place climate concern within a plural motivational field of health, taste, identity, convenience, and social norms.

A Slovenian cross-sectional survey provides the closest climate-linked evidence for meat-intake outcomes. The full sample comprised 2,990 participants. The study analyzed school-enrolled youth by educational stage and 799 secondary-school students by educational track. In the school-enrolled-youth models, climate attitudes were associated with current meat intake, retrospectively reported reduction over the previous 3 years, and reduction intention. Significant statistical indirect effects through climate attitudes appeared for tertiary-vs.-secondary stage comparisons across all three outcomes and for selected secondary-track comparisons of past reduction and intention (6). These cross-sectional indirect-effect patterns define targets for longitudinal evaluation.

Qualitative studies add detail to this motivational field. Interviews with 15–16-year-olds identified limited knowledge of the planetary-health benefits of plant-based diets, low food-preparation skills, and modest interest despite confidence that dietary change was possible (7). Adolescents discussing insect protein described favorable sustainability attitudes together with reluctance, naturalness concerns, and social influence (8). In a survey of 8,799 children and adolescents aged 10–19 years, health was the leading stated dietary motive overall, with motives differing by dietary pattern and sex (9). Across these studies, climate-related meaning is embedded in taste, health, identity, convenience, competence, and perceived normality.

Available climate-motive studies concentrate in adolescent samples. Adolescence often provides expanded opportunities to articulate values and participate in food decisions, while actual control continues to vary across households and institutions. Figure 1 represents age-related agency as a context operating across the outcome pathway.

**Figure 1:**
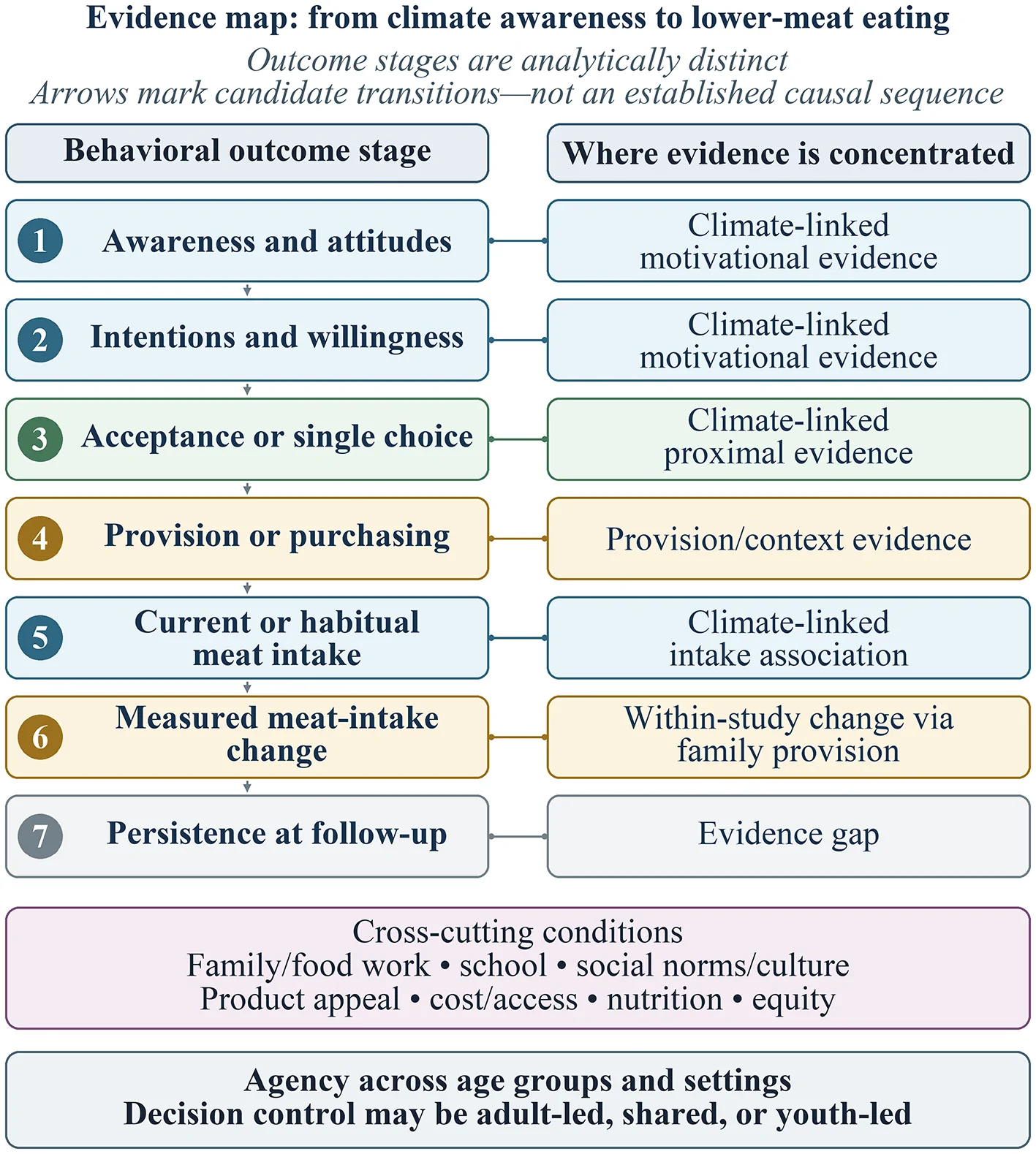
Evidence map of behavioral outcome stages and evidence concentration.

### 3.2 Climate-linked proximal behavioral evidence

One preregistered study comprising two randomized message experiments placed sustainability communication closest to immediate choice and intention. Among adolescents aged 12–19 years, educational messages improved experiment-reported sustainable food choices and intentions relative to unrelated messages; peer-norm content added motivational value, while dynamic and static norm messages performed similarly (10). The experiments establish an immediate response to message content within the study setting and identify peer norms as one mechanism through which information can become actionable.

### 3.3 Family decision-making, provision, and measured intake

Family research shifts attention from personal motivation to the organization of food decisions. In a survey of parents of children aged 5–8 years, parental meat attachment was associated with meal choices alongside perceived taste, health, balance, and child preference (11). In a French community, 49% of parents supported a second weekly vegetarian school meal and 26% supported daily provision, with support patterned by education, household diet, and food-choice motives (12). These studies show how adult preferences and material routines define the choices available to children.

Observed and qualitative family studies further identify negotiation as a mechanism. In a parent-adolescent decision study, particular child and dyadic behaviors were associated with a lower final meat proportion even though adolescents did not generally exert more influence than parents (13). Research with Chinese families living in New Zealand described parents as change agents who reorganized meals, rebuilt trust, and encouraged shared responsibility during meat reduction (14). Adult-led, shared, and youth-led decisions can therefore coexist within the same developmental period and household.

The clearest within-study meat-intake change comes from provision-based intervention. A six-week randomized trial supplied Danish families with three weekly insect- or plant-based dinners. Diet records indicated modest reductions in meat protein while estimated total protein intake was maintained; replacement was smaller among children and remained below the intended 20% target (15). The intervention manipulated household provision, locating its behavioral leverage in food availability and preparation. A four-month single-arm family mHealth program focused on plant proteins and food waste achieved high retention and satisfaction, with preliminary self-reported behavioral signals concentrated mainly among mothers (16). This evidence stream positions provision, household food work, and shared implementation as direct routes to intake change and as complementary targets for climate-oriented communication.

### 3.4 School and product conditions

Adjacent food-education studies identify cooking participation, sensory learning, preparation, and social identity as contextual supports for willingness and acceptance. A school-based vegan cooking workshop was followed by increases in pro-vegetarian social identity, attitudes, and dietary intentions, with peer-norm change associated with change in intention (17). A quasi-experimental canteen program reported improved knowledge and some willingness outcomes, with food-specific variation in liking (18). In Madagascar, children's stated willingness to eat farmed crickets increased from 16% before preparation-focused education to 87% afterward (19). These interventions clarify modifiable conditions near the intention-to-choice transition; the delivered activities included cooking, tasting, and preparation-focused education.

Sensory and service evidence is closer to the meal itself. Across 2,324 children aged 9–15 years in 12 European countries, liking, positive emotional responses, and lower food neophobia were associated with greater acceptance of sustainable school recipes and less food waste; pasta and legume-burger formats generally performed better than soups (20). Repeated meal ratings from a French city showed comparable liking of vegetarian and non-vegetarian main dishes (21). These studies identify familiarity, sensory quality, and recipe format as uptake conditions; total intake remains a distinct outcome.

Institutional implementation also depends on adults and social legitimacy. Parents' and teachers' support for school plant-based policies was associated with favorable norms, lower meat attachment, and perceived meal attributes (22). Swedish upper-secondary students' responses to a vegan school month revealed political and gendered meanings that shaped acceptance and resistance (23). Mixed-method evidence from Swedish canteens found that staff expectations of greater waste from vegetarian meals were not supported by measured waste data (24). A recipe-engineering study produced a high-organic, lower-meat school menu with an ingredient price 2.3% above baseline, a difference that was not statistically significant; labor, preparation, acceptance, and observed service costs were outside that model (25).

Product studies extend these conditions beyond school menus. Interviews with parent-child pairs found plant-based meat more familiar and acceptable than cultivated meat or insects, while curiosity coexisted with concerns about processing and naturalness (26). Dutch children aged 8–10 years were open to tasting plant-based analogs and could imagine requesting them at home, particularly when products resembled meat (27). Product acceptance therefore defines an opportunity for substitution; displacement of meat depends on subsequent purchasing, provision, repeated selection, and consumption.

### 3.5 Nutrition, affordability, and equity

Nutrition evidence defines the quality of the change being pursued. Analyses of planned primary-school meals show that vegetarian menus can markedly reduce modeled greenhouse-gas emissions while maintaining comparable overall nutritional quality under explicit menu standards (28). Linear-programming studies similarly demonstrate the mathematical feasibility of nutritionally adequate, climate-compatible adolescent diets through pattern-specific substitutions and fortified foods (29). Product audits show that many school meat substitutes provide fiber but are ultra-processed and may be high in salt, while meal planners balance environmental goals with familiarity and acceptability (30). Recent public-school menu analysis also identified environmental gains alongside modeled nutritional adequacy (31).

Evidence at the individual level reinforces the importance of replacement quality. A systematic review found potential nutrient inadequacies across dietary groups, with vitamin B12, iron, and zinc requiring particular attention in plant-based groups (32). Clinical data among children aged 5–10 years associated some plant-based dietary patterns with favorable adiposity or lipid profiles alongside lower bone-mineral measures and specific micronutrient risks; supplementation accounted for some vitamin B12 and vitamin D differences (33). Among teenage girls, vegetarian/vegan and pescatarian patterns were associated with a higher prevalence of iron deficiency than omnivorous diets (34). A prospective cohort found different nutrient and body-composition associations for healthier and less healthy plant-based diet indices, placing diet quality above a single dietary label (35).

Affordability and equity enter at the point where feasible menus must become available meals. Parent support for vegetarian school provision varied with education and existing household diet in one community (12), while the school-menu cost study measured ingredients rather than total labor or service costs (25). Cultural meanings also shaped meat rationalizations, family change, responses to vegan school provision, and perceptions of alternative proteins (5, 14, 23, 26). These findings identify access, food labor, cultural legitimacy, and distributional reach as pathway conditions that can be measured alongside selection, consumption, nutrition, and waste.

### 3.6 Developmental opportunities for dietary agency

Across studies, decision control varied by setting and participant role. Caregivers organized choice and provision in family studies involving younger children (11, 15), while schools structured exposure and opportunities in studies 18 and 19. Adolescent studies more often measured articulated motives, peer norms, or family negotiation (4, 5, 10, 13, 23). Parents remained central in the adolescent family-decision study (13); family-process research also identified parental implementation, trust, and shared responsibility at the household level (14). Younger children nevertheless expressed preferences or willingness (18, 19, 27). Together, these findings define dietary agency through who can influence the next decision, what resources are available, and whether participation changes provision or intake.

## 4 Discussion: implications for research and intervention design

### 4.1 Evidence-supported interpretation

The evidence map yields three principal findings. First, climate awareness is most clearly represented in motivational and proximal outcomes; one cross-sectional study also links climate attitudes with current intake, reported past reduction, and future intention among school-enrolled youth (6). Surveys and qualitative studies position environmental concern alongside health, taste, identity, convenience, and social norms, while message experiments demonstrate immediate responses in sustainable dietary choices and intentions. Second, the clearest within-study meat-intake change is concentrated in provision-focused family intervention. Third, family, school, product, cultural, nutritional, and material conditions act at multiple points across the evidence map. The resulting model links motivation with opportunities for food selection and repeated consumption while separating current status, change, and persistence.

These findings also sharpen the role of development. The reviewed literature contains more explicit climate-motive research among adolescents and more gatekeeper or provision research involving younger children. This distribution supports an opportunity-based interpretation: developmental change can expand participation in food decisions, but the effect of age depends on who controls purchasing, preparation, menus, and resources. Treating agency as a measured feature of the decision setting allows developmental hypotheses to be tested within, as well as between, age groups.

Table 1 summarizes the highest outcome observed in each evidence stream, its contribution to the evidence map, and priorities for testing.

**Table 1:** Evidence map of pathways and contextual conditions linking climate awareness with meat-related outcomes.

Evidence stream	Highest outcome observed	Contribution to the pathway	Implication for testing
Climate-related knowledge, attitudes, and motives (4, 5, 7–9)	Attitudes, intentions, current-action reports, and consumption-pattern associations	Locates climate concern within health, taste, identity, convenience, and social norms	Measure climate constructs with meat-specific repeated behavior and mediation pathways
Climate-linked intake associations (6)	Current intake, retrospective past reduction, and future intention at one survey occasion	Connects climate attitudes with meat-related outcomes in school-enrolled youth	Test temporal ordering with repeated measures and meat-specific outcomes
Climate-linked message experiment (10)	Immediate sustainable choice and intention	Identifies sustainability information and peer norms as proximal components	Link experimental exposure to repeated selection, amount consumed, and total meat intake
Food-education and experiential context (17–19)	Identity, knowledge, intention, willingness, and liking	Identifies cooking, tasting, preparation, and participation as contextual supports	Compare education alone with education plus choice and repeated availability
Family decision-making and provision (11–16)	Parental choice, dyadic meal choice, provision, and within-study meat-intake change	Shows how gatekeeping, negotiation, food work, and availability reach meal-level behavior	Estimate climate motivation and provision separately while measuring compensation and persistence
School provision and sensory context (20–25)	Acceptance, liking, observed waste, policy support, and modeled ingredient cost	Identifies recipe format, familiarity, social legitimacy, procurement, and service conditions	Connect what is offered and selected with intake, waste, total cost, and equitable reach
Alternative-protein acceptance (26, 27)	Perceptions, willingness to taste, and anticipated household requests	Defines product-level opportunities and barriers for substitution	Test whether acceptance produces repeated purchase, provision, consumption, and meat displacement
Nutrition, affordability, and equity (1, 28–35)	Modeled menu composition, nutrient intake or status, and environmental performance	Defines the nutritional and material quality of feasible lower-meat pathways	Prespecify replacement quality, supplementation, food security, culture, and distributional effects

### 4.2 Intervention hypotheses

For communication, the evidence supports testing climate information together with socially credible and feasible action. Messages can connect environmental benefits with taste, health, fairness, animal welfare, family identity, and culinary competence (5, 7–9), while peer-norm content can make a proposed action recognizable within adolescents' social worlds (10). Comparative trials can separate the contribution of information, norms, food skills, and actual availability and can measure whether immediate choices recur after the message is removed.

For families, observed gatekeeping and negotiation patterns provide a rationale for testing different distributions of responsibility. Caregiver-focused provision can be compared with shared planning or youth-led meal selection while holding food availability constant. Outcomes can include what was purchased and prepared, child participation, portion consumed, meat eaten elsewhere, nutritional adequacy, and persistence. Such designs would identify when climate-related motivation adds value beyond practical provision.

For schools and municipalities, existing studies identify components for factorial or sequential evaluation: curriculum, peer participation, tasting, familiar recipe formats, procurement standards, repeated service, and feedback. A lower-meat default represents one testable way to reduce friction, provided that offered meals, selection, intake, waste, cost, and equitable reach are measured separately. Linking classroom, kitchen, and dining-space data would reveal whether a program changes motivation, opportunity, or consumption.

Developmental comparisons can be embedded within these designs. In younger samples, caregiver- and institution-focused approaches can be compared with child-directed messages. In middle childhood, trials can test whether education gains strength when paired with tasting, preparation, and a genuine role in menu feedback. In adolescence, studies can test climate information with peer norms, food skills, and control over selection. Measuring family structure, culture, socioeconomic conditions, school policy, and food availability within each age group would identify when age-related opportunities for agency modify an intervention response.

### 4.3 Research priorities and review scope

A common outcome framework would substantially improve comparability. Studies can report climate knowledge and concern, attitudes, intention, willingness, acceptance, meal selection, amount consumed, total meat intake, compensation outside the intervention setting, and persistence as separate variables. Repeated measures and follow-up would locate where change is maintained or interrupted. Mediation analysis could then test whether climate-related motivation operates through norms, skills, perceived control, or availability.

Research also needs broader social and geographic coverage. Much of the evidence comes from European or other high-income settings. Studies in Madagascar and among Chinese migrant families show how product familiarity, food traditions, and feasible routes can differ (14, 19). In settings where animal-source foods address undernutrition, the appropriate sustainability objective may concern production, waste, or selective substitution rather than generalized reduction. Reporting socioeconomic position, food security, cultural background, gender, and control over purchasing and preparation would make distributional effects visible.

This Mini Review used English-language Articles and Reviews indexed in Web of Science or Scopus, with one reviewer conducting screening and evidence-role coding. Its targeted search maps heterogeneous pathways and contexts through full-text evidence cards, study-design descriptors, outcome-stage coding, and a complete retained evidence pool. The resulting audit trail identifies the transition from immediate response to meat-specific change and persistence as the central priority for longitudinal and intervention research.

## 5 Conclusion

The literature positions climate awareness as one motivational component of lower-meat eating among children and adolescents. Its clearest documented contribution lies in attitudes, intentions, willingness, immediate sustainable choices, and cross-sectional intake associations. A provision-based family trial provides the clearest within-study meat-intake change, whereas school and product studies chiefly identify conditions for acceptance, provision, and waste. Sensory quality, cultural legitimacy, affordability, nutrition, and access shape the feasibility and durability of acceptable lower-meat meals.

Development shapes opportunities to participate in food decisions as a heterogeneous, context-dependent process. Measuring food-decision control alongside age allows family, school, and peer processes to be evaluated as moderators across the evidence map. The next generation of studies can connect climate-related motivation with genuine opportunities to select, obtain, and repeatedly consume nutritionally adequate lower-meat meals, making current intake, within-study change, and persistence separately measurable.
